# The Clinical Relevance of Neurocognitive Measures in Addiction

**DOI:** 10.3389/fpsyt.2013.00185

**Published:** 2014-01-10

**Authors:** Reshmi Marhe, Maartje Luijten, Ingmar H. A. Franken

**Affiliations:** ^1^Institute of Psychology, Erasmus University Rotterdam, Rotterdam, Netherlands; ^2^Department of Child and Adolescent Psychiatry, VU University Medical Center, Amsterdam, Netherlands; ^3^Behavioural Science Institute, Radboud University Nijmegen, Nijmegen, Netherlands

**Keywords:** neurocognitive processes, addiction, substance relapse, clinical relevance, attentional bias, cognitive control

## Abstract

One of the major challenges in addiction treatment is relapse prevention, as rates of relapse following treatment remain very high across the main classes of drugs of abuse. Relapse prevention could be improved by a better understanding of the factors that influence treatment outcomes, including better predictors of risk of relapse following treatment. Recent developments in cognitive neuroscience point to neurocognitive measures (i.e., brain-imaging measures during cognitive-task performance) as potential predictors of relapse. These might even be better predictors than self-report measures, such as craving. We first give an overview of the current state of the field, and then discuss the outstanding challenges and future directions in this area of research.

Substance-dependent individuals often relapse, despite their efforts to stay abstinent (1). Substance dependence is therefore characterized as a chronic relapsing disorder (2, 3). For example, after 1–6 months follow-up, 40–80% of the heroin- and cocaine-dependent patients who were in treatment relapse (4–7). To improve treatment and treatment assignment for these patients it is important to gain knowledge about the psychological and biological processes underlying treatment outcome and relapse. The aim of this review is to describe the use of neurocognitive measures in addiction research in relation to the prediction of relapse, and discuss their clinical relevance. Since there is considerable overlap between the various substances of abuse, we attempt to focus on factors which are known to play a role in substance-use disorders in general (i.e., alcohol, cigarette smoking, stimulants, and opiates). Where research on a specific substance is described this is indicated.

## Predictors of Substance Relapse: From Self-Report to Neurocognitive Measures

Over the years, various kinds of predictors have been studied in relation to substance-use relapse such as demographic characteristics and other variables such as drug use severity, medical problems, and psychopathology [for reviews see Ref. (8, 9)]. In addition, self-report measures of emotional states such as negative affect (10), and drug-related states such as craving (11–13) have also found to be predictive of substance relapse [contrasting findings: (14, 15)]. However, an important limitation of using self-report measures is that people – and particularly substance-dependent individuals – may have low insight into their motivations and misrepresent their thoughts and feelings, or their reports may be biased due to social desirability (16, 17).

Neurocognitive measures, including neurophysiological measures such as functional magnetic resonance imaging (fMRI) and electroencephalography (EEG) during cognitive-task performance, arguably overcome some of the limitations of self-report measures. During neurocognitive assessments, participants are often unaware of the purpose of the assessment. Automatic, fast cognitive processes that are unavailable to conscious introspection can influence behavior [e.g., Ref. (18)]. These processes cannot be assessed via self-reports, but they can be assessed by neurocognitive psychological assessments. In the last two decades, the use of these neurocognitive assessments to examine neurobiological and cognitive processes underlying addiction has emerged in addiction research (19). Additionally, implicit cognitive and physiological measures hold some promise in predicting drug relapse and may even be better predictors than self-report measures [e.g., Ref. (13, 20–22)]; we will explore this possibility later in this review. Before elaborating upon the association between neurocognitive measures and substance relapse, a short overview of some relevant neurocognitive theories of addiction and supporting empirical evidence will be provided.

## Neurocognitive Processes in Addiction

Various recent theories of addiction suggest an imbalance in motivational and cognitive control processes in substance-dependent individuals (23–27). More specifically, it is proposed that substance-dependent individuals have an overactive motivational system that develops as a consequence of repetitive drug use. Repetitive drug use sensitizes the mesolimbic reward system up to a point that merely the perception (and not only the use) of drugs or drug cues becomes salient (28). Because of this incentive salience that is being attributed to drug-related stimuli, attention is automatically oriented to these stimuli, also referred to as attentional bias (24).

A wide range of behavioral studies have confirmed the presence of an attentional bias to substance cues in dependent individuals [for review see Ref. (29)] and its association with self-reported craving has also been supported (30). Accordingly, there has been much interest in investigating the neurobiological substrates of attentional bias. Recent fMRI studies showed that attentional bias to substance cues is associated with activity in prefrontal brain areas such as the anterior cingulate cortex (ACC) and dorsolateral prefrontal cortex (DLPFC) (31–36), other cortical areas including the insula (32, 35, 36) and also subcortical activation in the nucleus accumbens (34) and amygdala (35, 37). It has been suggested that these brain regions play a role in the imbalance between motivational and control processes; that is, the nucleus accumbens and amygdala are evidently involved in the bottom-up process of salience attribution to substance-related stimuli while at the same time top-down attentional resources of the prefrontal executive areas might be impaired or depleted when focusing on cognitive tasks in the presence of distracting drug-related cues (25, 38).

Other theories suggest that the ability to control drug use behaviors is reduced in drug-dependent individuals, particularly in conditions that deplete cognitive recourses, like craving or cue-exposure (38, 39). Several studies indeed reported that cognitive control processes, which have their neural basis in regions of the prefrontal cortex, are impaired in substance-dependent people (25, 40, 41). Two specific cognitive control processes (i.e., inhibitory control and error-processing) may be particularly involved in the continuation of substance use. Inhibitory control is crucial when one would like to control substance use by implementing the inhibition of inappropriate behavior, while error-processing is involved in monitoring ongoing behavior to prevent future mistakes. A recent review of neuroimaging studies (41) into inhibitory control and error-processing suggests that substance dependence is associated with reduced brain activation during inhibitory control and error-processing in the ACC, inferior frontal gyrus (IFG), and DLPFC. In addition, event-related potentials such as the N2 and error-related negativity (ERN), reflecting brain activation associated with inhibitory control and error-processing respectively, seem to be reduced in substance-dependent patients. These findings implicate that cognitive control processes of substance-dependent people may be dysfunctional, thereby contributing to the lack of control over substance-related behaviors.

In sum, both drug-related motivational processes as well as dysfunctions in cognitive control may contribute to compulsive drug use behavior and this might explain why substance-dependent individuals cannot control their drug use and often relapse after a period of abstinence (23, 38, 39). Below, we will discuss studies that have prospectively examined neurocognitive measures and their association with substance-use outcomes. Note that all studies described below report effects on group level; the clinical relevance for individual patients will be discussed later in this review.

## Neurocognitive Predictors of Treatment Outcome and Relapse

Well-established research on the role of cognitive and neurobiological processes in addiction has resulted in an increased focus on neurocognitive measures as predictors of treatment outcome and relapse. On the behavioral level, results have mainly showed an association between attentional bias and treatment outcome in substance dependency [although some results have been inconsistent; for a recent review see Ref. (42)]. To the best of our knowledge, only two fMRI studies have used an attentional bias paradigm to examine whether brain-activity related to attentional bias was associated with substance relapse (36, 43). Other fMRI studies have examined whether cue-reactivity to substance-related stimuli might predict substance-use outcomes [(44, 45), contrasting findings: (46); see Table 1].

**Table 1:** **Overview of studies described in the present review investigating neurocognitive predictors of substance relapse after treatment**.

Study	Participants	Task measures	Assessment time	Outcome measures	Main results and analysis		Main conclusion
**ATTENTIONAL BIAS/CUE-REACTIVITY**							
Kosten et al. (44)	17 Cocaine dependents	Passive viewing of cocaine-related (3-min) and neutral video (60-s)	During a 2-week in-patient stay prior to a 10-week outpatient clinical trial	Proportion of all cocaine-negative urines during 10-week outpatient clinical trial;	↑	fMRI contrast: first 30-s of cocaine tape vs. 60-s of neutral tape;	Increased brain activation during cocaine cue-exposure in precentral gyrus, posterior cingulate, superior temporal gyrus, lingual gyrus, and inferior occipital gyrus was associated with lower proportion of urines testing negative for cocaine. Relapse was associated with higher posterior cingulate activation (extended anteriorly) during cue-exposure.
				Relapsers vs. non-relapsers (verified by urine tests).	↑	Correlations between outcome measures and contrasted brain-activity.	
Heinz et al. (46)	12 Detoxified alcohol dependents	Passive viewing of alcohol-related and neutral pictures and positive and negative pictures	1-Week after 3-week detoxification program	Alcohol intake during 6-month follow-up period (biweekly assessment of alcohol intake using form 90).	0	fMRI contrast: alcohol vs. neutral stimuli;	Brain activation elicited by briefly presented alcohol-associated stimuli vs. neutral stimuli was not associated with relapse to alcohol intake.
						Correlations between outcome measures and contrasted brain-activity.	
Janes et al. (37)	21 Smokers	Passive viewing of smoking and neutral pictures while occasionally responding to prompt animal pictures (to avoid study fatigue)	Pre-smoking cessation treatment	Lapse vs. abstinence during 8-week smoking cessation (weekly self-reports verified by breath tests).	↑	fMRI contrast: smoking vs. neutral stimuli;	Lapsers had increased brain activation for smoking-related vs. neutral stimuli in the insula, ACC, posterior cingulate cortex, amygdala, primary motor cortex, premotor cortex, inferior parietal cortex, parahippocampal gyrus, thalamus, putamen, cerebellar hemispheres and vermis, prefrontal cortex, and striate and extrastriate cortex;
						Correlations between outcome measures and contrasted brain-activity;	
					↓	Functional connectivity analyses in lapsers vs. abstainers;	Lapsers had reduced connectivity in an insula-containing network and dACC;
					↑	Discriminant analysis.	A prediction model including behavioral Stroop effect and anterior insula and dACC activation to smoking-related vs. neutral stimuli predicted outcomes with 79% accuracy.
Beck et al. (45)	46 Detoxified alcohol dependents	Passive viewing of alcohol-related, neutral and scrambled pictures	1-Week after detoxification treatment	Relapsers vs. abstainers during 3-month follow-up (biweekly assessment of alcohol intake using form 90).	↑	fMRI contrast: alcohol vs. neutral stimuli;	Increased brain activation in the left medial prefrontal cortex during processing of alcohol-related stimuli is associated with relapse and not with abstinence. In contrast, increased brain activation in the right ventral tegmental area and left and right ventral striatum during processing of alcohol-related stimuli is associated with abstinence and not with relapse.
					↑^a^	Correlations between outcome measures and contrasted brain-activity.	
Marhe et al. (43)	26 Cocaine dependents	Drug Stroop task (cocaine words, neutral words, and letter strings)	First week of detoxification treatment	Number of days of cocaine use in the last 30 days (assessed at 3-month follow-up, verified by urine test).		fMRI contrast: cocaine vs. neutral stimuli;	Increased attentional bias-related activity in the dACC was associated with more days of cocaine use at 3-month follow-up;
					↑	Linear regression with regions of interest (involved in attentional bias) as predictor variables and number of days of cocaine use as dependent variable.	Both dACC-activity and self-reported craving accounted for 45% in explained variance (unique contribution was respectively 23 and 22%).
**COGNITIVE CONTROL/EXECUTIVE FUNCTIONS**							
Paulus et al. (53)	46 Meth-amphetamine dependents	2-Choice prediction task	1-Month after in-patient treatment	Self-reported relapse vs. non-relapse within 1-year follow-up; Self-reported time to relapse (by means of structured interview).	↓ ↓	fMRI contrast: choice prediction vs. simple response;Stepwise discriminant function analysis with the areas of differences between relapsers and non-relapsers as predictor variables and relapse status as dependent variable;Stepwise Cox regression analysis.	Relapse was associated with reduced activity in right insula, right posterior cingulate, and right middle temporal gyrus during the 2-choice prediction task. Brain-activity in these regions predicted relapse with 94% sensitivity and 86% specificity; Time to relapse was best predicted by low activation in right middle frontal gyrus, right middle temporal gyrus, and right posterior cingulate cortex activation.
Brewer et al. (21)	20 Cocaine dependents	Classic Stroop task	Pre-clinical treatment trail	Proportion of cocaine-negative urines; Self-reported longest abstinence from cocaine (days);	↑ ↑	fMRI contrast: incongruent Stroop trials vs. congruent Stroop trials;	Brain regions involved in cognitive control are differentially associated with specific treatment outcomes; cocaine free urines was associated with hyperactivity in the right putamen. Self-reported abstinence was associated with hyperactivity in the left posterior cingulate cortex and left ventromedial prefrontal cortex. Treatment retention was associated with hypoactivity in de DLPFC. Treatment retention was also associated with behavioral Stroop interference.
				Weeks in treatment.	↓	Correlations between outcome measures and contrasted brain-activity.	
Marhe et al. (22)^b^	49 Cocaine dependents	Eriksen flanker task	First week of detoxification treatment	Number of days of cocaine use in the last 30 days (assessed at 3-month follow-up, verified by urine test).	↓	Linear regression with ERN amplitude as predictor variable and number of days of cocaine use as dependent variable.	Reduced ERN amplitude (indicating diminished error-processing) was associated with more days of cocaine use at 3-month follow-up;
							A prediction model including substance-use severity and self-reported craving accounted for 33% of explained variance (ERN was only significant individual predictor; unique contribution was 7%).
Luo et al. (54)	97 Cocaine dependents	Stop signal task	2–4 Weeks after residential treatment	Cocaine use at 14, 30, 60, and 90 days after discharge (assessed with timeline-follow back method on substance-use calendar, verified by urine tests).	↓	fMRI contrast: stop error vs. stop success trials; Logistic and Cox regressions.	Brain-activity related to error-processing in the dACC was associated with relapse in men and women. Reduced activity in the thalamus and insula was a gender specific (resp. women, men) predictor of relapse. Receiver operating characteristic curve was 0.85.

*0 = no association between neurocognitive measures and outcome; ↑ = elevated levels of cognition/brain-activity is associated with worse treatment outcome or relapse; ↓ = reduced levels of cognition/brain-activity is associated with worse treatment outcome or relapse*.

*^a^Elevated brain-activity was associated with abstinence*.

*^b^EEG study*.

*ACC, anterior cingulate cortex; dACC, dorsal anterior cingulate cortex; DLPFC, dorsolateral prefrontal cortex; ERN, error-related negativity*.

Overall, the cue-reactivity studies show that enhanced brain-activity during substance cue-exposure in prefrontal, sensory, motor, and limbic (sub)cortical areas is associated with substance relapse [(44, 45), cf. (46)]. Note that results in alcohol dependent patients are inconsistent. Heinz et al. (46) found no relation between neural cue-reactivity and alcohol intake after treatment. In contrast, Beck et al. (45) found that increased prefrontal brain-activity (during passive viewing of alcohol cues) was associated with relapse after treatment while increased activity in the ventral tegmental area and ventral striatum were associated with abstinence after treatment, indicating that different brain processes (cognitive control vs. reward system) are differently associated with treatment outcome in alcohol dependents.

In smokers, Janes et al. (36) found that both behavioral attentional bias for smoking-related words (measured with a Stroop task outside of the scanner) along with reactivity of the brain to smoking cues were predictive of smoking relapse. In addition, anterior insula and dorsal ACC (dACC) activation strongly correlated with respectively larger interference of drug-related words and low accuracy during the Stroop task, suggesting that these regions might represent the neural correlates of attentional bias that may be important for identifying individuals at risk of relapse. This idea is supported by a recent study showing that in cocaine-dependent patients, increased dACC-activity related to attentional bias for cocaine stimuli (measured with a drug Stroop task) was associated with relapse to cocaine use after treatment (43). Thus, it seems that the dACC – involved in salience detection and conflict monitoring (47–50) – plays an important role in relapse risk. It has been suggested that hyperactivity in the dACC reflects enhanced conflict in the presence of emotionally salient distracters, such as substance-related stimuli. Hence, increased dACC-activity in response to substance cues might reflect that patients at risk of relapse need more top-down resources to focus on cognitive tasks when substance-related cues are present as distractors during the task. This implies that relapse-vulnerable individuals have a reduced ability to control their substance-related cognitions, regulated by the dACC, and consequently might experience difficulties in controlling their substance-use behavior.

Studies examining the association between cognitive control processes (e.g., inhibitory and attentional control, behavior monitoring) and relapse following treatment have generally found that impaired cognitive functioning is associated with a higher risk of relapse [for recent reviews see Ref. (51, 52)]. Only a few studies have examined whether brain-activity related to performance on cognitive tasks is associated with substance relapse. Paulus and colleagues (53) were the first to report that brain-activity during a simple two-choice task (measured with fMRI) can predict relapse in methamphetamine dependence. This indicated that relapse vulnerability was associated with reduced activation in a brain network related to decision-making (e.g., DLPFC, parietal, temporal, and insular cortex). An fMRI study in cocaine-dependent patients found that behavioral interference on the classical Stroop task and associated brain activations in prefrontal and striatal regions are predictive of treatment outcome, which indicates that impaired attentional control may be a marker of relapse risk (21). Two recent studies have examined the association between brain-activity during error-processing and relapse in cocaine-dependent patients [(22, 54), see also Ref. (55)]. These studies found that reduced brain-activity during error-processing is associated with cocaine use after treatment. More specifically, Luo et al. (54) found that reduced thalamus, insula, and dACC-activity, measured with fMRI, was predictive of relapse to cocaine. Marhe et al. (22) examined the ERN, an event-related potential reflecting the automatic detection of an error. Results showed that ERN amplitudes are associated with increased cocaine use 3 months after detoxification treatment. These findings suggest that underactive error-related brain-activity might be a marker of relapse risk (55).

Most of the abovementioned studies indicate that cognitive and motivational processes are associated with relapse vulnerability. This is only a first step toward clinical implementation, and current findings need to be interpreted with caution since group results are not necessarily valid on individual results. Different methodologies and designs hamper a direct comparison between these studies. Further, it is not known whether the results found in one substance-dependent group (e.g., cocaine-dependent patients) can be generalized to other substances. In addition, the treatment settings are quite diverse. However, if these findings will stand after replication and provide more accurate information on individual-level prediction, they might eventually help to identify substance-dependent patients that are at risk of relapse into substance use. Obviously, this is not only the case for neurocognitive predictors, but for all predictors of relapse, including demographical, self-report, or behavioral measures.

## Are Neurocognitive Measures Better Predictors of Relapse than Self-Report Measures?

It is clear that neurocognitive measures such as fMRI and – to a lesser degree – EEG are relative expensive and time consuming, limiting their daily use in clinical practice. Therefore, in order to advocate the use of neurocognitive measures in clinical practice, there should be a clear advantage compared to inexpensive and more feasible self-report predictors of relapse such as self-reported craving and substance-use severity. One hypothetical advantage could be that neurocognitive measures explain relapse better than self-report measures – or at least explain additional variance in predicting relapse over and above self-report measures.

Current relapse prediction studies addressing motivational and cognitive control processes provide some preliminary indications for this. Some of the studies addressing motivational aspects found that brain-activity during substance cue-exposure was associated with relapse, whereas self-reported craving and substance-use severity were not associated with relapse (44–46). Marhe et al. (43) also found that the association between attentional bias-related brain-activity and relapse persisted when controlling for self-reported substance-use severity. However, self-reported craving and attentional bias-related brain-activity contributed equally to the prediction of cocaine relapse (i.e., craving explained 23% and dACC-activity explained 22% of the variance). In addition, studies addressing cognitive control also show additional benefit of neurocognitive measures above self-reported measures. Paulus and colleagues (53) reported that substance-use severity was not associated with methamphetamine relapse, while brain-activity during decision-making was. Brewer et al. (21) showed that brain activation during Stroop interference was more strongly related to treatment outcome than self-reported craving. Additionally, another study (22) showed that brain-activity during error-processing was a unique predictor of cocaine relapse, over and above substance-use severity and self-reported craving.

Hence, some relapse prediction studies indicate that neurocognitive measures might make a unique contribution to the prediction of relapse and may even be able to better predict outcomes than self-report measures such as craving. In addition, regarding the specific role of subjective craving, results suggest that the relationship between neurocognitive measures and relapse is not accounted for or mediated by craving [e.g., Ref. (42)]. Speculatively, they reflect two processes that might both explain variance in relapse risk.

## Limitation Concerning the Use of Neurocognitive Measures in Clinical Practice and Suggestions for Future Research

Neurocognitive methods provide us with crucial information of how brain responses are related to clinical outcomes. Although there are some indications that neurocognitive measures could be relevant in clinical practice there are some issues that need further research before these measures can be applied in a clinical setting. Obviously, one of the biggest challenges is moving from group-level associations with treatment outcomes/relapse to individual-level prediction of such outcomes, for example by using receiver operating characteristic analyses (53, 54). This technique provides information on the sensitivity and specificity of predictors, which need to be sufficient enough to contribute to treatment planning for an individual patient.

In the long run, neurocognitive techniques such as fMRI could be used to investigate individual risk profiles for example with the use of machine-learning approaches (56). However, in the short term it is not feasible and cost-effective to scan every patient in substance-use treatment programs using fMRI. Although the same problems are true for EEG, it is arguably a more cost-effective and more accessible neuroimaging tool that could be implemented in treatment programs more easily. The idea to use EEG as a diagnostic instrument has gained interest specifically for ERP components that have adequate psychometric properties, such as the ERN (57, 58). Future studies should be carried out to examine whether routine assessment of for example ERN amplitudes in cocaine-dependent patients [see Ref. (22)] could identify patients vulnerable for relapse. Additionally, treatment programs could be tailored to the patient’s need to improve outcomes. For example, by providing specific training programs to improve cognitive [e.g., Ref. (59)] and/or brain functions [e.g., Ref. (60)].

Another limitation is that all studies use different measures of treatment outcome/relapse (e.g., self-reported use or abstinence, urine screens, time to relapse) which makes it difficult to compare the present results. Using the same (multiple) outcome measures across studies would certainly advance the field, also beyond neurocognitive studies.

Also, current prediction studies using neurocognitive measures have not addressed the role of response inhibition, which is another important index of cognitive control. Some evidence comes from cross-sectional studies showing that ex-substance users have increased brain-activity during inhibitory control (assessed with Go-Nogo) compared to current users, suggesting that response inhibition might underlie recovery from substance dependence [smokers: (34); cocaine users: (61)]. Future studies should address the predictive role of response inhibition in prospective designs.

Finally, it is of theoretical as well as clinical importance that studies investigating the predictive value of neurocognitive processes of addiction include self-report and behavioral measures in prediction models. It is important to test whether the unique contribution of these relatively “new” measures is high enough in addition to well-established, more easily administered measures. Ideally, relapse prediction models should be multifactorial and should include (socio)demographic, psychological, physiological, and cognitive variables. Of course, large sample sizes are needed to accomplish sufficient power (62).

## Conclusion

Neuroimaging research has yielded important information on neurocognitive mechanisms of substance dependence in relation to treatment processes and outcome. Results have shown that neurocognitive measures can provide information on relapse vulnerability over and above the information gained from self-report measures such as craving on a group level. However, regarding clinical utility it is important that all prediction studies report the sensitivity and specificity of neurocognitive relapse predictors. This will allow gaining more knowledge on the suitability of neurocognitive measures for individual risk taxation, necessary for implementation in clinical settings.

## Conflict of Interest Statement

The authors declare that the research was conducted in the absence of any commercial or financial relationships that could be construed as a potential conflict of interest.
